# Mortality and Attrition Rates within the First Year of Antiretroviral Therapy Initiation among People Living with HIV in Guangxi, China: An Observational Cohort Study

**DOI:** 10.1155/2021/6657112

**Published:** 2021-02-10

**Authors:** Jinhui Zhu, Mohammed Adnan Yousuf, Wenmin Yang, Qiuying Zhu, Zhiyong Shen, Guanghua Lan, Yi Chen, Huanhuan Chen, Wensheng Fan, Hui Xing, Yiming Shao, Yuhua Ruan, Liming Li

**Affiliations:** ^1^Guangxi Center for Disease Control and Prevention, Nanning, China; ^2^Department of Public Health, Western Kentucky University, Bowling Green, KY, USA; ^3^Department of Health Services Administration, Florida International University, Miami, FL, USA; ^4^State Key Laboratory of Infectious Disease Prevention and Control (SKLID), Collaborative Innovation Center for Diagnosis and Treatment of Infectious Diseases, Chinese Center for Disease Control and Prevention (China CDC), Beijing, China; ^5^School of Public Health, Peking University, Beijing, China

## Abstract

**Objective:**

To assess the mortality and attrition rates within the first year of antiretroviral therapy (ART) initiation among people living with human immunodeficiency virus (PLHIV) in rural Guangxi, China.

**Design:**

Observational cohort study. *Setting*. The core treatment indicators and data were collected with standard and essential procedures as per the Free ART Manual guidelines across all the rural health care centers of Guangxi. *Participants*. 58,115 PLHIV who were under ART were included in the study. *Interventions*. The data collected included sociodemographic characteristics that consist of age, sex, marital status, route of HIV transmission, CD4 cell count before ART, initial ART regimen, level of ART site, and year of ART initiation. *Primary and Secondary Outcome Measures*. Mortality and attrition rate following ART initiation.

**Results:**

The average mortality rate was 5.94 deaths, and 17.52 attritions per 100 person-years within the first year of ART initiation among PLHIV. The mortality rate was higher among intravenous drug users (Adjusted Hazard Ratio (AHR) 1.27, 95% Confidence Interval (CI) 1.14-1.43), prefecture as a level of ART site (AHR 1.14, 95% CI 1.02-1.28), and county as the level of ART site (AHR 2.12, 95% CI 1.90-2.37). Attrition was higher among intravenous drug users (AHR 1.87, 95% CI 1.75-2.00), the first-line ART containing AZT (AHR 1.09, 95% CI 1.03-1.16), and first-line ART containing LVP/r (AHR 1.34, 95% CI 1.23-1.46).

**Conclusion:**

The mortality and attrition rates were both at the highest level in the first year of post-ART; continued improvement in the quality of HIV treatment and care is needed.

## 1. Introduction

HIV infection in China began as a drug-driven epidemic among injection drug users, and with time, it shifted to the mainly sexual route [1, 2]. By the end of 2015, a total of 577,423 individuals were living with HIV in China [3]. It is estimated that new cases of HIV epidemic scale up to 50,000 individuals annually [4], irrespective of China's National Free Antiretroviral Treatment Program (NFATP) that began in 2003 [5]. NFATP is a community-based public health program that helped to reduce the mortality rate among HIV-infected individuals in China [6, 7]. It develops in three phases. Initiation was from 2003 to 2005. The threshold for the NFATP in 2003 was CD4+ T cell counts less than 200 cells/*μ*L. [8] The first scale-up was from 2005 to 2008. The ART threshold was raised to CD4+ T cell counts less than 350 cells/*μ*L, and further scale-up and standardization were from 2009 to 2011. It rapidly expanded the treatment for prevention from 2012 to 2015; ART has been initiated regardless of CD4+ T cell counts [9–11].

According to Tang et al., by 2015 in China, 382,139 PLHIV have received Antiretroviral Therapy (ART) [12]. This rapid expansion of ART has further resulted in a drastic reduction in mortality among HIV-infected individuals, while this mortality rate remains much higher than in developed countries [13, 14]. Nevertheless, some HIV-infected patients might not have been aided by ART because of the limited access to care in resource-limited areas and also attrition of the patients after ART initiation [15, 16]. Thus, attrition during ART regimen is associated with progression of HIV to AIDS, treatment failure, drug resistance, and even mortality [4]. Although there are achievements by China's NFATP, high-quality evidence is needed to better understand mortality and attrition rates within the early year of ART initiation among Chinese PLHIV that can help with continuing improvements in the quality of HIV treatment and care.

Guangxi is located on the southern coast of China. The reported numbers of HIV cases in Guangxi are estimated to be 10% of the total reported cases in China, which is the highest number of HIV cases in China [1, 17]. The objective of this research is to assess the mortality and attrition rates within the first year of ART initiation among PLHIV in Guangxi.

## 2. Materials and Methods

### 2.1. Patient and Public Involvement

Since the study is retrospective, patients or the public were not involved in the design or in the conduct of the study.

### 2.2. Study Design and Study Population

This observational cohort was conducted in the rural areas of Guangxi, in southwest China. The cohort profile and characteristics of this database have been previously described [8, 10, 16]. The data were collected from 2003 to 2015. The eligibility criteria for the participants were 18 years or older, HIV-positive patients, recipient of first-line ART at any point between 2003 and 2015, willingness, and those who provide written consent for the study. It has 64% of participants still in follow-up. The study was approved by the institutional review board of the Guangxi Center for Disease Control and Prevention (reference number for the ethics committee to this manuscript is GXIRB2016-0047).

Attrition was defined as a cessation of ART or loss to follow-up as recorded in the HIV treatment database [18]. Lost to follow-up was defined as missing more than 90 days after the last date seen in the clinic, which was also defined as the date of withdrawal [10, 12].

### 2.3. Treatment Regimen and Criteria

China recommended first-line HAART regimen as per the WHO guidelines. First-line HAART regimens before 2008 consisted of (zidovudine (AZT) or stavudine (D4T)) + (didanosine (DDI) or lamivudine (3TC)) + (nevirapine (NVP) or efavirenz (EFV)) [8, 9]. Zidovudine, stavudine, didanosine, and nevirapine are generically produced in China and were standard prescriptions since the start of the program in 2003, whereas lamivudine and efavirenz are branded drugs that became available in 2005 [19] and gradually became more commonly prescribed following preferential use in those with strong adverse reactions to the former standard regimens. The first-line ART regimen in the second edition National Free ART Guideline in 2008 was revised to consist of (tenofovir (TDF) or azidothymidine (AZT)) + lamivudine (3TC) + (efavirenz (EFV) or nevirapine (NVP)). The NFATP treatment criteria included CD4+ T cell counts less than 200 cells/*μ*L from 2003 to 2008, CD4+ T cell counts less than 350 cells/*μ*L from 2008 to 2012, and starting 2012, ART has been initiated irrespective of CD4+ T cell counts. According to the recent WHO guidelines, ART is recommended immediately for all HIV infected patients irrespective of CD4 cell count [20]. Before treatment initiation, PLHIV were given free education and counseling on the importance of ART and adherence. The initial treatment regimen and criteria could be associated with treatment effects [9].

### 2.4. Data Collection

Variables of the PLHIV who received ART included baseline characteristics of age, sex, marital status, route of HIV transmission, CD4 cell count before ART, initial ART regimen, level of ART site, and year of ART initiation. HIV infection status was determined by an enzyme immunoassay (EIA) and an HIV-1/2 Western blot confirmation. It did not include self-reported. Treating physicians who provided the ART at the local medical centers managed the case reports forms at the time of initiating ART and follow-up at 0.5, 1, 2, and 3 months and every 3 months afterwards. CD4 cell count was assessed at each follow-up. The case report forms were uploaded into a web-based database hosted by CDC, China. Follow-up variables were evaluated every 3 months after ART and included death or attrition. Variables collected at each follow-up included transferals to another clinic, cessation of ART, loss to follow-up, duration of ART, and survival status, which were collected from hospital records or having clinic doctors call family members to inquire about death. The date of death was based on death certificate information. The core treatment indicators and data were collected with a standard and essential procedures as per the Free ART Manual guidelines across all the health care centers.

### 2.5. Statistical Analysis

An observational cohort study was conducted using the Guangxi data from the NFATP. The outcomes of interest (mortality and attrition rates) following the first-line ART regimen drugs D4T, AZT, TDF, and LPV/r were obtained. Mortality and attrition rate cumulative incidence was calculated on the basis of Poisson distributions and as deaths and attrition per 100 person-years of follow-up. Time zero was defined as the date of ART initiation, and data was censored on June 30, 2016. Patients were censored if they were still alive or transferred to another clinic for care.

Cox proportional hazard models were used to evaluate treatment effects of initial ART regimen on death and attrition (cessation of ART or loss to follow-up) of the first year in PLHIV who started ART between 2003 and June 2015, respectively. Competing risks for cause-specific hazard models were censored accordingly. Cause-specific Cox proportional hazard models were used to assess the effects of CD4+ count levels before ART on attrition and death. To control for potential confounding, the following baseline variables were included: age, sex, marital status, route of HIV infection, initial ART regimen, level of ART site, and year of ART initiation. Variables were combined and analyzed with the Statistical Analysis System software (SAS 9.4™ for Windows; SAS Institute Inc., Cary, NC, USA). *P* value <0.05 was regarded as statistically significant, and all tests were 2 sided. The results were presented with the adjusted hazard ratios (AHRs) and at 95% CI.

## 3. Results

### 3.1. Characteristics

Of the 58,270 PLHIV who initiated ART between January 2003 and June 2015 in Guangxi, China, 147 patients were less than 18 years of age, and 8 patients had no any follow-up data after ART, so they were excluded from the study. A total of 5,822 patients within the first year of ART initiation lost to follow-up, and 3,125 patients stopped ART. Of the 3,125 patients who stopped ART, 1,244 (39.8%) were due to poor adherence, 1,059 (33.9%) were due to drug side effects, 65 (2.1%) were due to economic issues, and 757 (24.2%) were due to other reasons. Finally, a total of 58,115 eligible patients were included in this cohort study.

Table 1 shows the general characteristics of these patients. The majority (33.2%) of the individuals belong to the 35-49 years age group, 67.0% were males, and 69.2% were married or cohabitation. Of all the participants with sexually transmitted infection, 85.4% self-reported as heterosexual intercourse, 1.6% self-reported as homosexual, 10.2% received through intravenous drug use, and 2.8% as other routes. The CD4 cell count before initiation of ART was less than 200 cells/*μ*L for 59.8% individuals followed by 200-350 cells/*μ*L for 28.0%, 350-500 cells/*μ*L in 7.8%, equal to or more than 500 in 2.8%, and missing 1.5%. Under the initial ART regimen, the first-line ART containing D4T constitute 24.5%, ART containing AZT was 39.7%, ART containing TDF was 24.0%, ART containing LPV/r was 7.8%, and others were 4.0%. Levels of ART site as provincial were 18.8%, the prefecture was 38.8%, and the county was 42.4%. Because the threshold for the NFATP was changed, more and more PLHIV were involved in ART. The number of patients initiating ART raised from 13.3% during the years 2003-2008 to 56.1% during 2012-2015.

### 3.2. Mortality Rates

Among all the HIV-infected participants who started ART between 2003 and 2015, there are 5,377 deaths; the average mortality rate was 2.96 deaths per 100 person-years among all participants. The mortality rate per 100 person-years was 5.94 in the first year of ART, and then, it decreased thereafter (Supplementary Table 1).  Table 2 shows the mortality and attrition rates in PLHIV started ART between 2012 and 2015 in Guangxi.

Univariate Cox regression analyses indicated that several factors were significantly associated with mortality rate, including age, sex, marital status, route of HIV infection, CD4 cell count (cells/*μ*L) before ART, initial ART regimen, level of ART site, and year of ART initiation. In the multivariate model, factors associated with lower risk of mortality rate were female gender (AHR 0.55, 95% CI 0.50-0.60), homosexual intercourse as HIV transmission route (AHR 0.50, 95% CI 0.28-0.88), CD4 count 200-350 before initiation of ART (AHR 0.26, 95% CI 0.23-0.30), CD4 count 350-500 (AHR 0.19; 95% CI 0.14-0.25), CD4 count ≥500 (AHR 0.17, 95% CI 0.10-0.27), the first-line ART containing AZT (AHR 0.53, 95% CI 0.49-0.59), the first-line ART containing TDF (AHR 0.70, 95% CI 0.62-0.79), other drugs (AHR 0.40, 95% CI 0.32-0.50), and year of ART initiation from 2009 to 2011 (AHR 0.77, 95% CI 0.70-0.86) and from 2012 to 2015 (AHR 0.49, 95% CI 0.43-0.55). The following are the factors associated with a higher risk of mortality rate: age group 35-49 years old (AHR 1.40, 95% CI 1.26-1.55), age group 50-70 years old (AHR 1.95, 95% CI 1.74-2.17), and equal to or more than 70 years (AHR 2.70, 95% CI 2.31-3.15); other marital status (AHR 1.20, 95% CI 1.11-1.30); route of HIV transmission—intravenous drug use (AHR 1.27, 95% CI 1.14-1.43); prefecture as the level of ART site (AHR 1.14, 95% CI 1.02-1.28); and county as the level of ART site (AHR 2.12, 95% CI 1.90-2.37) (Table 3). Similar study results were observed even after excluding individuals with missing baseline CD4 counts and PLHIV who started ART between 2003 and June 2011, respectively (Supplementary Table 2, Supplementary Table 3, and Supplementary Table 4).

### 3.3. Attrition Rates

Among all the HIV-infected participants who started ART between 2003 and 2015, 15,744 attritions were reported. The average attrition rate was 8.66 per 100 person-years among all participants. The attrition rate per 100 person-years was 17.52 in the first year of ART, and then, it decreased thereafter (Supplementary Table 2).

Univariate Cox regression analyses indicated that age, sex, marital status, route of HIV infection, CD4 cell count (cells/*μ*L) before ART, initial ART regimen, level of ART site, and year of ART initiation were significantly associated with attrition rate. In the multivariate model, factors associated with lower risk of attrition rate were female gender (AHR 0.89, 95% CI 0.85-0.94), homosexual intercourse as HIV transmission route (AHR 0.45, 95% CI 0.35-0.58), the first-line ART containing TDF (AHR 0.81, 95% CI 0.75-0.87), and other drugs (AHR 0.87, 95% CI 0.76-1.00). The following are factors associated with a higher risk of attrition rate: age group 35-49 years old (AHR 1.15, 95% CI 1.09-1.22), followed by age group 50-70 years old (AHR 1.55, 95% CI 1.46-1.64), equal to or more than 70 years (AHR 2.51, 95% CI 2.30-2.73), other marital status (AHR 1.32, 95% CI 1.26-1.38), route of HIV transmission—intravenous drug use (AHR 1.87, 95% CI 1.75-2.00), CD4 count 350-500 before initiation of ART (AHR 1.11, 95% CI 1.03-1.21), CD4 count ≥500 (AHR 1.29, 95% CI 1.15-1.45), the first-line ART containing AZT (AHR 1.09, 95% CI 1.03-1.16), ART containing LPV/r (AHR 1.34, 95% CI 1.23-1.46), and year of ART initiation from 2009 to 2011 (AHR 1.74, 95% CI 1.60-1.91) and from 2012 to 2015 (AHR 1.90, 95% CI 1.73-2.08).  Table 4. Similar study results were observed even after excluding individuals with missing baseline CD4 counts (Supplementary Table 6 and Supplementary Table 7).

## 4. Discussion

With time, China's ART guidelines evolved as per the WHO recommendations [21]. And hence, this is one of the reasons in our study; we see a drastically high number of participants during the period 2012-2015 of ART initiation, besides increased awareness regarding treatment, and a high incidence of new HIV cases is reported annually [4].

The overall mortality rate was 2.96 per 100 person-years in HIV-infected patients who started ART from January 2003 to June 2015. It was at the peak of 5.94 per 100 person-years during the first year, lower than a Tanzanian study [22], and gradually reduced to 0.63 per 100 person-years at the end of the 11^th^ year of initiation of ART. The impact of ART has significantly reduced the mortality among PLHIV in this study. The scale-up and standardization of NFATP after 2008 has shown to be very successful in lowering the mortality rate in China [23]. Furthermore, the rapid expansion and treatment for prevention from 2012 had significantly lowered the mortality rate. However, according to WHO, the mortality rate is higher during the early months of ART as early adverse drug reactions, opportunistic infections, and/or immune reconstitution inflammatory syndrome may develop during that period. These complications are common in patients who initiate ART with advanced HIV, existing coinfections, malnourished individuals, very low CD4 cell counts, severely low hemoglobin, and low body mass index [24, 25]. However, some PLHIV might die early after receiving ART, due to several very sick and no complications.

The mortality rate of the first year in PLHIV who started ART is higher among aged patients. This is similar to a previous study conducted by HIV-Casual Collaboration [26]. (Supplementary Table 2). Older individuals have a poorer immunologic response and other aging-related problems like hypertension, hypertriglyceridemia, lipodystrophy, and low bone mineral density [27]. Also, females have a lower mortality rate than males after ART initiation [28, 29].(Supplementary Tables 2 and 3). Previous studies have also demonstrated that there is a lower mortality rate among females after initiation of ART. It could be possibly attributed to better responses to drugs, early diagnosis, adherence to ART, and psychosocial factors in females [30]. Thus, interventions should be designed which are aimed at giving extra support to the groups with high mortality. The CD4 cell counts below 200 cells/*μ*L resulted in high mortality. Previous studies have shown a relationship among persons with CD4 cell counts below 200 cells/*μ*L developing drug-resistant viral strains that resulted in a higher risk of mortality [31]. Initiating ART at CD4 count cell ≥ 500 can have a favorable outcome in reducing mortality [24, 32]. (Supplementary Table 4).

Mortality is also found to be high in county-level of ART sites than the provincial sites. This is supported by the fact that county and prefecture-level health care providers have cited adverse reaction, opportunistic infections, and other complications that resulted in the referral to the provincial centers [33]. The health care system at the county level in China has inadequate resources and few specialty doctors. The Chinese government is managing these inequalities in the distribution of health care. They are providing financial and professional incentives, rural training and recruitment, and administrative measures as intervention strategies to tackle disparities in health care [34]. The National Health Commission is providing incentives for doctors for treating HIV/AIDS-affected individuals and also providing fellowship that encourages specialists to provide care to patients and clinical training to allied health care workers in rural areas [33, 35]. In Guangxi, HIV cases are diagnosed late, and also, there is a delay in the initiation of free ART, due to the essential steps needed in providing care [7]. Simple steps in early diagnosing and initiating ART should be implemented that can help in early diagnosis of HIV, initiating treatment, and reducing the mortality rate.

The overall attrition rate was 8.66 per 100 person-years in HIV infected patients who started ART between January 2003 and June 2015. In most of the attrition, more than 56% occurred during the first year of ART initiation. A high attrition rate can result in suboptimal treatment results, an increase in mortality rate, increased cost of care, and further transmission of HIV [15]. A previous study conducted on ART in China has shown side effects from the treatment, patient self-request, and adherence issues as the top three reasons for attrition during ART [18]. It is also worthwhile to note that patients who were lost to follow-up might have different treatment outcomes compared to patients who remained in the study, such as high mortality and viral load rebound due to stop ART [36]. Nevertheless, a high attrition rate is worrisome and needs urgent attention. Attrition was high as the age increases, and men were more likely than females to experience attrition within the first year of ART initiation (Supplementary Table 6). Also, men have poor health-seeking behaviors than women that lead to more advanced disease when initiating ART. This adds to more mortality in men than in their counterparts [37]. The risk of attrition was also high among those individuals whose marital status was other than married or cohabitation, intravenous route of HIV transmission, county-level of ART site, and the year of ART initiation between the years 2012 and 2015.

The study has shown that both mortality and attrition are high among intravenous drug users than the sexual route. Past studies have shown that patients identified as intravenous drug users are less likely to seek care after HIV diagnosis and more likely to miss scheduled appointments, thus resulting in the loss in follow-up [38, 39] and attrition. Attrition in the county is higher than provincial and prefecture sites. Previous studies have demonstrated that attrition is high among peripheral health clinics than urban clinics, possibly due to the lack of capacity to manage complicated HIV cases [40]. The attrition rate is less among patients with low CD4 cell count (Supplementary Table 7), possibly because patients with lower CD4 cell counts are more likely to have AIDS symptoms; such individuals may have better compliance to ART [12].

In our study, the mortality rate was high in persons with first-line ART containing D4T followed by TDF. The attrition rate was high in persons with first-line ART containing LPV/r followed by AZT. Past studies have shown that patients in resource-limited countries had first-line ART regimen failure [41]. However, patients who switch early to booster protease inhibitors (LPV/r) containing regimen have shown the effectiveness of the treatment due to early viral suppression. Since the majority of these patients reside and seek ART in rural areas (prefecture and county) that had no prior exposure to protease inhibitors [42], the viral suppression depends on the adherence. A large study in South Africa has shown that PLHIV without prior exposure to protease inhibitors had shown that viral failure with boosted protease inhibitors is barely coupled with the surfacing of new protease mutations [43]. Since in our study the attrition is high among persons with first-line ART containing LPV/r, we can conclude that lack of adherence to protease inhibitors is a primary cause of high attrition. Also, the past studies have well documented gastrointestinal side effects during the initial therapy with LPV/r than the other protease inhibitors [44, 45]. This can, in turn, lead to high attrition among the LPV/r users. Future studies should be directed towards the areas with resource-limited settings that should perform population-level adherence to protease inhibitors as part of the nationwide treatment programs and also use of other protease inhibitors with better gastrointestinal tolerability and less adverse effects. Continued improvement in the quality of HIV treatment and care is needed to further reduce the mortality rate and attrition rate in Chinese patients.

There are a few limitations to the study. Firstly, some patients in the study were missing CD4^+^ cell count data, which is an important risk factor for attrition. There is a likelihood that the missing CD4^+^ cell count data will differ from the patients with complete data. To compensate this, a missing CD^+^ cell count category has been added to the analysis. Secondly, there is likely bias due to residual confounding. Many potential confounding variables could not be included in the cause-specific Cox proportional hazard models because the data was collected at the time of diagnosis of HIV, which is much later than the disease occurrence. Only a few variables like age, gender, and route of HIV transmission were unchanged at the time of HIV infection; hence, they can be included in the analysis. Thirdly, the study outcomes included death and attrition (cessation of ART or loss to follow-up). The reasons for attrition of cessation of ART and loss to follow-up are different. Death might be unreported, due to high attrition of loss to follow-up. Also, we need to note that the data collected from 2003 are the reflection of real-world treatment realities in a resource-limited setting. Such observational data can have possible inherent biases.

In conclusion, China's ART has clearly demonstrated reduced HIV mortality and attrition over the years. The mortality and attrition rates were both at the highest level in the first year of post-ART and declined year by year. So it is very important to strengthen the PLHIV management before ART and the first year post-ART. Additionally, with the constantly increasing of PLHIV's CD4 level before ART, it is necessary to focus on their management to avoid attrition. As the NFATP is expanding in rural areas, intensive efforts are needed to tackle the inequalities in health care, improve and increase drug supply, and ensure the proper training of health care workers and availability of specialists to treat PLHIV. Continued improvement in the quality of HIV treatment and care is needed to further reduce the mortality rate and attrition rate in Chinese patients.

## Figures and Tables

**Table 1:** Characteristics of PLHIV who started ART between 2003 and June 2015 in Guangxi, China.

Variable	Number	%
Total	58,115	—
Age (years)		
18-34	18,358	31.6
35-49	19,290	33.2
50-70	17,457	30.0
≥70	3,010	5.2
Sex		
Male	38,957	67.0
Female	19,158	33.0
Marital status		
Married or cohabitation	40,224	69.2
Other	17,891	30.8
Route of HIV transmission		
Heterosexual intercourse	49,608	85.4
Intravenous drug use	5,954	10.2
Homosexual intercourse	925	1.6
Other	1,628	2.8
CD4 cell count (cells/*μ*L) before ART		
<200	34,774	59.8
200-350	16,252	28.0
350-500	4,550	7.8
≥500	1,648	2.8
Missing	891	1.5
Initial ART regimen		
The first-line ART containing D4T	14,244	24.5
The first-line ART containing AZT	23,059	39.7
The first-line ART containing TDF	13,965	24.0
The first-line ART containing LPV/r	4,510	7.8
Other	2,337	4.0
Level of ART site		
Provincial	10,920	18.8
Prefecture	22,525	38.8
County	24,670	42.4
Year of ART initiation		
2003-2008	7,741	13.3
2009-2011	17,800	30.6
2012-2015	32,574	56.1

ART: antiretroviral therapy; D4T: stavudine; AZT: azidothymidine; TDF: tenofovir; LPV/r: ritonavir-boosted lopinavir; HR: hazard ratio; AHR: adjusted hazard ratio.

**Table 2:** Mortality and attrition rates in PLHIV started ART between 2012 and June 2015 in Guangxi, China, by year post-ART initiation.

Variable	Number	Person-years	Deaths	Deaths per 100 person-years (95% CI)	Attritions	Attritions per 100 person-years (95% CI)
Total	32,574	68,463.57	1,913	2.79 (2.67-2.92)	7,438	10.86 (10.62-11.10)
Year post-ART initiation						
The first year	32,574	28,659.32	1,221	4.26 (4.03-4.49)	5,440	18.98 (18.49-19.47)
The 2nd year	26,046	24,937.71	417	1.67 (1.52-1.83)	1,490	5.97 (5.68-6.27)
The 3rd year	16,274	15,815.86	190	1.20 (1.03-1.37)	564	3.57 (3.28-3.85)
The 4th year	8,861	8,689.38	83	0.96 (0.75-1.16)	201	2.31 (2.00-2.62)

**Table 3:** Mortality rate of the first year in PLHIV who started ART between 2003 and June 2015 in Guangxi, China.

Variable	Number	Deaths	Person-years	Deaths per 100 person-years (95% CI)	HR (95% CI)	*P*	AHR (95% CI)	*P*
Total	58,115	3,035	51,066.90	5.94 (5.74-6.15)	—	—	—	—
Age (years)								
18-34	18,358	684	16,591.47	4.12 (3.82-4.42)	Reference		Reference	
35-49	19,290	974	17,079.86	5.70 (5.35-6.05)	1.38 (1.25-1.52)	<0.001	1.40 (1.26-1.55)	<0.001
50-70	17,457	1,091	15,016.71	7.27 (6.84-7.69)	1.74 (1.58-1.91)	<0.001	1.95 (1.74-2.17)	<0.001
≥70	3,010	286	2,378.87	12.02 (10.66-13.38)	2.80 (2.44-3.21)	<0.001	2.70 (2.31-3.15)	<0.001
Sex								
Male	38,957	2,481	33,799.71	7.34 (7.06-7.62)	Reference		Reference	
Female	19,158	554	17,267.19	3.21 (2.95-3.47)	0.44 (0.40-0.48)	<0.001	0.55 (0.50-0.60)	<0.001
Marital status								
Married or cohabitation	40,224	1,976	35,698.49	5.54 (5.30-5.77)	Reference		Reference	
Other	17,891	1,059	15,368.41	6.89 (6.49-7.30)	1.23 (1.14-1.33)	<0.001	1.20 (1.11-1.30)	<0.001
Route of HIV transmission								
Heterosexual intercourse	49,608	2,494	43,780.65	5.70 (5.48-5.91)	Reference		Reference	
Intravenous drug use	5,954	440	4,994.46	8.81 (8.01-9.61)	1.52 (1.37-1.68)	<0.001	1.27 (1.14-1.43)	<0.001
Homosexual intercourse	925	12	866.24	1.39 (0.62-2.15)	0.25 (0.14-0.44)	<0.001	0.50 (0.28-0.88)	0.016
Other	1,628	89	1,425.56	6.24 (4.98-7.51)	1.10 (0.89-1.36)	0.391	1.03 (0.83-1.28)	0.774
CD4 cell count (cells/*μ*L) before ART								
<200	34,774	2,622	30,201.91	8.68 (8.36-9.01)	Reference		Reference	
200-350	16,252	283	14,605.50	1.94 (1.72-2.16)	0.22 (0.20-0.25)	<0.001	0.26 (0.23-0.30)	<0.001
350-500	4,550	48	4,076.29	1.18 (0.85-1.50)	0.14 (0.10-0.18)	<0.001	0.19 (0.14-0.25)	<0.001
≥500	1,648	16	1,459.20	1.10 (0.57-1.62)	0.13 (0.08-0.21)	<0.001	0.17 (0.10-0.27)	<0.001
Missing	891	66	724.00	9.12 (6.97-11.26)	1.03 (0.81-1.31)	0.824	1.21 (0.95-1.55)	0.129
Initial ART regimen								
The first-line ART containing D4T	14,244	1,347	1,2276.66	10.97 (10.40-11.54)	Reference		Reference	
The first-line ART containing AZT	23,059	791	20,389.98	3.88 (3.62-4.14)	0.35 (0.32-0.39)	<0.001	0.53 (0.49-0.59)	<0.001
The first-line ART containing TDF	13,965	547	12,508.72	4.37 (4.02-4.73)	0.40 (0.36-0.44)	<0.001	0.70 (0.62-0.79)	<0.001
The first-line ART containing LPV/r	4,510	263	3,765.83	6.98 (6.16-7.81)	0.63 (0.55-0.72)	<0.001	1.02 (0.88-1.18)	0.837
Other	2,337	87	2,125.71	4.09 (3.25-4.93)	0.38 (0.31-0.47)	<0.001	0.40 (0.32-0.50)	<0.001
Level of ART site								
Provincial	10,920	475	9,810.33	4.84 (4.42-5.27)	Reference		Reference	
Prefecture	22,525	953	20,001.78	4.76 (4.47-5.06)	0.98 (0.88-1.09)	0.718	1.14 (1.02-1.28)	0.020
County	24,670	1,607	21,254.79	7.56 (7.20-7.92)	1.54 (1.39-1.71)	<0.001	2.12 (1.90-2.37)	<0.001
Year of ART initiation								
2003-2008	7,741	656	6,865.09	9.56 (8.84-10.27)	Reference		Reference	
2009-2011	17,800	1,158	15,542.50	7.45 (7.03-7.87)	0.77 (0.70-0.85)	<0.001	0.77 (0.70-0.86)	<0.001
2012-2015	32,574	1,221	28,659.32	4.26 (4.03-4.49)	0.44 (0.40-0.49)	<0.001	0.49 (0.43-0.55)	<0.001

ART: antiretroviral therapy; D4T: stavudine; AZT: azidothymidine; TDF: tenofovir; LPV/r: ritonavir-boosted lopinavir; HR: hazard ratio; AHR: adjusted hazard ratio.

**Table 4:** Attrition rate of the first year in PLHIV who started ART between 2003 and June 2015 in Guangxi, China.

Variable	Number	Attritions	Person-years	Attritions per 100 person-years (95% CI)	HR (95% CI)	*P*	AHR (95% CI)	*P*
Total	58,115	8,947	51,066.90	17.52 (17.17-17.87)	—	—	—	—
Age (years)								
18-34	18,358	2,424	16,591.47	14.61 (14.04-15.18)	Reference		Reference	
35-49	19,290	2,770	17,079.86	16.22 (15.63-16.81)	1.11 (1.05-1.17)	<0.001	1.15 (1.09-1.22)	<0.001
50-70	17,457	2,958	15,016.71	19.7 (19.01-20.39)	1.35 (1.28-1.42)	<0.001	1.55 (1.46-1.64)	<0.001
≥70	3,010	795	2,378.87	33.42 (31.15-35.68)	2.27 (2.09-2.46)	<0.001	2.51 (2.30-2.73)	<0.001
Sex								
Male	38,957	6,284	33,799.71	18.59 (18.14-19.04)	Reference		Reference	
Female	19,158	2,663	17,267.19	15.42 (14.85-15.99)	0.83 (0.79-0.87)	<0.001	0.89 (0.85-0.94)	<0.001
Marital status								
Married or cohabitation	40,224	5,697	35,698.49	15.96 (15.55-16.36)	Reference		Reference	
Other	17,891	3,250	15,368.41	21.15 (20.44-21.86)	1.32 (1.27-1.38)	<0.001	1.32 (1.26-1.38)	<0.001
Route of HIV transmission								
Heterosexual intercourse	49,608	7,357	43,780.65	16.80 (16.43-17.18)	Reference		Reference	
Intravenous drug use	5,954	1,289	4,994.46	25.81 (24.43-27.18)	1.53 (1.44-1.62)	<0.001	1.87 (1.75-2.00)	<0.001
Homosexual intercourse	925	66	866.24	7.62 (5.83-9.41)	0.45 (0.36-0.58)	<0.001	0.45 (0.35-0.58)	<0.001
Other	1,628	235	1,425.56	16.48 (14.43-18.54)	0.98 (0.86-1.12)	0.783	1.08 (0.95-1.23)	0.226
CD4 cell count (cells/*μ*L) before ART								
<200	34,774	5,030	30,201.91	16.65 (16.21-17.10)	Reference		Reference	
200-350	16,252	2,598	14,605.50	17.79 (17.12-18.45)	1.07 (1.02-1.12)	0.006	1.01 (0.96-1.06)	0.765
350-500	4,550	783	4,076.29	19.21 (17.90-20.52)	1.15 (1.07-1.24)	<0.001	1.11 (1.03-1.21)	0.007
≥500	1,648	321	1,459.20	22.00 (19.65-24.34)	1.32 (1.18-1.48)	<0.001	1.29 (1.15-1.45)	<0.001
Missing	891	215	724.00	29.70 (25.83-33.57)	1.78 (1.55-2.04)	<0.001	1.62 (1.41-1.86)	<0.001
Initial ART regimen								
The first-line ART containing D4T	14,244	1,922	12,276.66	15.66 (14.97-16.34)	Reference		Reference	
The first-line ART containing AZT	23,059	3,845	20,389.98	18.86 (18.28-19.44)	1.20 (1.14-1.27)	<0.001	1.09 (1.03-1.16)	0.003
The first-line ART containing TDF	13,965	1,935	12,508.72	15.47 (14.80-16.14)	0.99 (0.93-1.05)	0.734	0.81 (0.75-0.87)	<0.001
The first-line ART containing LPV/r	4,510	1,004	3,765.83	26.66 (25.05-28.27)	1.70 (1.57-1.83)	<0.001	1.34 (1.23-1.46)	<0.001
Other	2,337	241	2,125.71	11.34 (9.94-12.73)	0.73 (0.64-0.83)	<0.001	0.87 (0.76-1.00)	0.044
Level of ART site								
Provincial	10,920	1,470	9,810.33	14.98 (14.24-15.73)	Reference		Reference	
Prefecture	22,525	3,378	20,001.78	16.89 (16.33-17.44)	1.13 (1.06-1.20)	<0.001	1.02 (0.96-1.08)	0.593
County	24,670	4,099	21,254.79	19.29 (18.71-19.86)	1.29 (1.21-1.36)	<0.001	1.04 (0.98-1.11)	0.178
Year of ART initiation								
2003-2008	7,741	694	6,865.09	10.11 (9.38-10.84)	Reference		Reference	
2009-2011	17,800	2,813	15,542.50	18.1 (17.45-18.75)	1.78 (1.64-1.93)	<0.001	1.74 (1.60-1.91)	<0.001
2012-2015	32,574	5,440	28,659.32	18.98 (18.49-19.47)	1.87 (1.73-2.02)	<0.001	1.90 (1.73-2.08)	<0.001

ART: antiretroviral therapy; D4T: stavudine; AZT: azidothymidine; TDF: tenofovir; LPV/r: ritonavir-boosted lopinavir; HR: hazard ratio; AHR: adjusted hazard ratio.

## Data Availability

All data supporting the findings of this study are available within the paper and its supplementary information file. The raw data are not publicly available due to governmental regulation.

## Abbreviations

D4T: Stavudine
AZT: Azidothymidine
TDF: Tenofovir
LPV/r: Ritonavir-boosted lopinavir
AHR: Adjusted hazard ratio
HR: Hazard ratio.

## Additional Points

*Strengths and Limitations of This Study*. (i) This is an observational cohort study in Guangxi, China, including 58,115 PLHIV. (ii) The large cohort provides strong evidence in support of our study results. (iii) Treatment effects of death and attrition were assessed through Cox proportional hazard models. (iv) This study might not be representative of all PLHIV in China.

## Supplementary Materials

Supplementary Materials Supplementary figure 1. Enrollment, and Follow-up of the Study Participants. Supplementary table 1. Mortality rates of patients with HIV who started ART between 2003 and June 2015 in Guangxi, China. Supplementary table 2. Mortality rates of the first year in patients with HIV who started ART between 2003 and June 2015 in Guangxi, China. Supplementary table 3. Mortality rates of the first year in patients with HIV who started ART between 2012 and June 2015 in Guangxi, China. Supplementary table 4. Mortalityrates of the first year in patients with HIV who started ART between 2003 and June 2015 in Guangxi, China, by CD4 count before ART. Supplementary table 5. Mortality rates of patients with HIV who started ART between 2003 and June 2015 in Guangxi, China. Supplementary table 6. Attrition rates of the first year in patients with HIV who started ART between 2003 and June 2015 in Guangxi, China. Supplementary table 7. Attrition rates of the first year in patients with HIV who started ART between 2003 and June 2015 in Guangxi, China, by CD4 count before ART.
