# Supplementary material for: Mortality and Attrition Rates within the First Year of Antiretroviral Therapy Initiation among People Living with HIV in Guangxi, China: An Observational Cohort Study
Source: Biomed Res Int. 2021 Feb 10;2021:6657112. doi: 10.1155/2021/6657112 (PMC7892219; doi:10.1155/2021/6657112)
Supplement: Supplementary Materials — Supplementary figure 1. Enrollment, and Follow-up of the Study Participants. Supplementary table 1. Mortality rates of patients with HIV who started ART between 2003 and June 2015 in Guangxi, China. Supplementary table 2. Mortality rates of the first year in patients with HIV who started ART between 2003 and June 2015 in Guangxi, China. Supplementary table 3. Mortality rates of the first year in patients with HIV who started ART between 2012 and June 2015 in Guangxi, China. Supplementary table 4. Mortalityrates of the first year in patients with HIV who started ART between 2003 and June 2015 in Guangxi, China, by CD4 count before ART. Supplementary table 5. Mortality rates of patients with HIV who started ART between 2003 and June 2015 in Guangxi, China. Supplementary table 6. Attrition rates of the first year in patients with HIV who started ART between 2003 and June 2015 in Guangxi, China. Supplementary table 7. Attrition rates of the first year in patients with HIV who started ART between 2003 and June 2015 in Guangxi, China, by CD4 count before ART. [file 6657112.f1.docx]

**Supplementary material**

Supplementary figure 1. Enrollment, and Follow-up of the Study Participants.

Supplementary table 1.Mortality rates of patients with HIV who started ART between 2003 and June 2015 in Guangxi, China

Supplementary table 2. Mortality rates of the first year in patients with HIV who started ART between 2003 and June 2015 in Guangxi, China

Supplementary table 3. Mortality rates of the first year in patients with HIV who started ART between 2012 and June 2015 in Guangxi, China

Supplementary table 4. Mortalityrates of the first year in patients with HIV who started ART between 2003 and June 2015 in Guangxi, China, by CD4 count before ART

Supplementary table 5.Mortality rates of patients with HIV who started ART between 2003 and June 2015 in Guangxi, China

Supplementary table 6. Attrition rates of the first year in patients with HIV who started ART between 2003 and June 2015 in Guangxi, China

Supplementary table 7. Attrition rates of the first year in patients with HIV who started ART between 2003 and June 2015 in Guangxi, China, by CD4 count before ART
